# SDHA loss of function mutations in a subset of young adult wild-type gastrointestinal stromal tumors

**DOI:** 10.1186/1471-2407-12-408

**Published:** 2012-09-14

**Authors:** Antoine Italiano, Chun-Liang Chen, Yun-Shao Sung, Samuel Singer, Ronald P DeMatteo, Michael P LaQuaglia, Peter Besmer, Nicholas Socci, Cristina R Antonescu

**Affiliations:** 1 Departments of Pathology, Memorial Sloan-Kettering Cancer Center, 1275 York Avenue, New York, NY, 10065, USA; 2 Institute Bergonie, Bordeaux, France; 3 Departments of Surgery, Memorial Sloan-Kettering Cancer Center, New York, NY, USA; 4 Developmental Biology, Sloan-Kettering Institute, New York, NY, USA; 5 Bioinformatics, Memorial Sloan-Kettering Cancer Center, New York, NY, USA; 6 Visiting Research Fellow from the Bergonie Institute, Bordeaux, France

**Keywords:** GIST, Pediatric, Wild-type, SDHA, SDHB, Succinate dehydrogenase complex II

## Abstract

**Background:**

A subset of KIT/PDGFRA wild-type gastrointestinal stromal tumors (WT GIST) have been associated with alteration of the succinate dehydrogenase (SDH) complex II function. A recent report identified four non-syndromic, KIT/PDGFRA WT GIST harboring compound heterozygous or homozygous mutations in SDHA encoding the main subunit of the SDH complex II.

**Methods:**

Next generation sequencing was applied on five pediatric and one young adult WT GIST, by whole exome capture and SOLiD 3-plus system sequencing. The putative mutations were first confirmed by Sanger sequencing and then screened on a larger panel of 11 pediatric and young adult WT GIST, including 5 in the context of Carney triad.

**Results:**

A germline p.Arg31X nonsense SDHA mutation was identified in one of the six cases tested by SOLiD platform. An additional p.D38V missense mutation in SDHA exon 2 was identified by Sanger sequencing in the extended KIT/PDGFRA WT GIST patients cohort. Western blotting showed loss of SDHA expression in the two cases harboring SDHA mutations, while expression being retained in the other WT GIST tumors. Results were further confirmed by immunohistochemistry for both SDHA and SDHB, which showed a concurrent loss of expression of both proteins in SDHA-mutant lesions, while the remaining WT tumors showed only loss of SDHB expression.

**Conclusions:**

Germline and/or somatic aberrations of SDHA occur in a small subset of KIT/PDGFRA WT GISTs, outside the Carney’s triad and are associated with loss of both SDHA and SDHB protein expression. Mutations of the SDH complex II are more particularly associated with KIT/PDGFRA WT GIST occurring in young adults. Although pediatric GIST consistently display alterations of SDHB protein expression, further molecular studies are needed to identify the crucial genes involved in their tumorigenesis.

## Background

The majority of gastrointestinal stromal tumors (GIST) harbor gain-of-function mutations in KIT or PDGFRA, resulting in the activation of the downstream pathways PI3K/AKT, Ras/MAPK, and JAK/STAT3, and playing a crucial role in tumorigenesis [1,2]. A subset of GIST lack specific KIT or PDGFRA mutations and form a heterogeneous group, including NF1, Carney Triad (CT), Carney-Stratakis Syndrome (CSS), pediatric and young adult GIST, and a small proportion (<10%) of sporadic adult GIST [3-8]. The mechanisms involved in the tumorigenesis of GIST lacking KIT or PDGFRA mutations are still poorly understood. A subgroup of these GISTs forms a unique clinicopathological entity, defined by negative staining for SDHB in addition to exhibiting distinct morphologic and clinical features [9,10]. Indeed, such SDH-deficient GISTs account for 5–7.5% of all gastric GISTs in unselected populations and include the great majority of pediatric GISTs. They are characterized by defects in cellular respiration and activation of pseudohypoxia signalling pathways [11]. The succinate dehydrogenase protein complex II (SDPC II) catalyzes the oxidation of succinate. SDHB is one of four protein subunits forming succinate dehydrogenase, the other three being SDHA, SDHC and SDHD. Loss of SDHB expression results in inhibition of the degradation of Hypoxia Inducing Factors (HIF), which in turn impairs apoptosis, and induces angiogenesis and glycolysis [12-15]. Loss of SDHB expression was first identified in GIST occurring in the context of Carney triad and in a subset of pediatric and adult GISTs with similar characteristics [9]. Loss of SDHB is also seen in WT GIST occuring in the context of CSS with genlius mutation of SDHB or SDHC (II). However, such mutations were also found in about 10% of sporadic GIST lacking KIT or PDGFRA mutation [11]. The mechanisms involved in loss of SDHB expression in SDH-deficient GIST without an associated SDHB or SDHC mutations remain unclear. One possible explanation is loss of function mutations in the SDHA gene, which have been recently identified in four patients (one pediatric and 3 young adult) with sporadic GIST lacking KIT or PDGFRA mutations [16,17]. The aim of this study was to assess globally by next generation sequencing mutations in the SDH-pathway, as well as determine the mutational and expression status of SDHA in a series of syndromic and sporadic GIST without mutations in KIT or PDGFRA.

## Methods

### Patients

Samples from 17 patients with gastric GIST selected on the basis of wild-type status for the KIT and PDGFRA genes were included for analysis. In 6 cases DNA from frozen tissue was available for next generation sequencing; in the remaining cases DNA was available from paraffin embedded material. Thirteen cases were diagnosed in children (≤18 years) and four cases in young adults (defined as older than 18 but younger than age of 30; 3 females and 1 male). Among the 13 pediatric cases (11 females, 2 males), five were diagnosed in the context of Carney’s triad (CT). The mean age at diagnosis for pediatric patients and young adults was 12 (range 8–18) and 23 years old (range 21–26), respectively. In all cases, KIT (exons 9, 11, 13, and 17), PDGFRA (exon 12, 14, 18) and BRAF (exon 15) genotyping was performed as previously described [18]. The clinicopathologic and genotype findings of seven of the pediatric and two of the young adult GIST cases have been previously described [19]. The study was approved by the Institutional Review Board (IRB-protocol 02–060).

### Sequencing by Oligonucleotide Ligation and Detection (SOLiD) and variant detection

Six cases, including five children, one of whom in the context of CT, and one young adult were analyzed by the SOLiD^TM^ (Sequencing by Oligonucleotide Ligation and Detection) platform. This next generation sequencing technology interrogates two bases at a time by ligation chemistry and detection of one of four colors associated with those specific two bases. Whole exome capture was performed on 1–3 μg of high quality genomic DNA using the SureSelect Human All Exon Kit, which targets 38 MB of exonic sequences, according to the protocol provided by the manufacturer (Agilent, Santa Clara, CA, USA). Enriched DNA libraries are then sequenced on a SOLiD 3plus system (Applied Bio-systems, Carlsbad, CA, USA), generating 68 million reads (50 bp). 86% of the targeted region was sequenced at a 10x-coverage.

The colorspace CSFASTA and QUAL files were first converted to double encoded FASTQ files which are then mapped to the target genome (hg19) using BWA with default options plus the colorspace mode option (−c). The output SAM files are tagged with read group ids merge across runs for the same library and then process with MarkDuplicates from Picard. Overlapping paired reads are resolved to remove redundant sequence. Then all the BAM for all samples are merged and process through the GATK toolkit to first Realign in/dels and the base Q scores are recalibrated with Recalibration tool. Paired samples (tumor/normal) are processed in pairs by muTect and we also run the entire cohort through the GATK Unified Genotyper to call both SNPs and in/dels. The variant output file (the VCF file) from the Unified Genotyper was then annotated using the SNPeff program with the UCSC RefSeq HG19 database to annotated the effect of the mutation. The raw output contained approximately 38,000 events. We then used a fairly stringent set of criteria to filter these calls. Only calls marked "PASS" be the Unified Genotype were retained all other events were filtered out. Further we removed events that had a non-reference allele frequency (NRAF) less than 10%. We also then removed any events that were annotated in dbSNP (v132) and removed those not annotated as HIGH IMPACT by SNPeff. This lead to a list of five events. For all 5 events we manually inspected the read pileups in IGV to look for possible artifacts. Of the 5 only one had no obvious defects. The other 4 had either strand bias issues and/or position bias (the variant reads tended to show the variants at the 3' end of the reads).

### Targeted exon resequencing

The mutational status of SDHA (exon 2, 9 and 13) was assessed by direct Sanger sequencing of genomic DNA. Protocols and primers are available on request. Sequence analysis was performed with Applied Biosystems Sequence ScannerTM v1.0.

### Western blotting

Western blotting was performed to assess the expression of SDHA in WT GIST with and without SDHA mutation. Frozen tissue from seven WT GIST (two with SDHA mutation and five without SDHA mutation) were homogenized in RIPA buffer supplemented with protease and phosphatase inhibitors. Electrophoresis and immunoblotting were performed on the protein extracts using 30 μg of protein per sample and the anti-SDHA rabbit polyclonal antibody (Cell Signaling Technology, Danvers, MA, USA) was diluted according to the manufacturers’ recommendations. Following hybridization with the secondary anti-rabbit antibody (Santa Cruz Biotechnology, Santa Cruz, CA, USA), the blots were incubated with Immun-Star horseradish peroxidase luminal/enhancer (Bio-Rad, Hercules, CA, USA) and exposed onto Kodak Biomax MR Film (Eastman Kodak Co., Rochester, NY, USA).

### Immunohistochemistry

Immunohistochemistry (IHC) was applied on all 17 WT GIST cases tested using SDHA (Abcam, Cambridge, UK, 1:1000) and SDHB (Sigma-Aldrich, St Louis, MO, 1:500), according to manufacturer’s recommendations. The results of the SDHA and SDHB immunohistochemistry were recorded blindly to the KIT, PDGFRA or SDHA genotyping. One KIT exon 11 mutant GIST from a young adult patient was used as control. The immunoreactivity was scored as negative (loss of expression) if the tumor cells were negative but the entrapped normal tissues (endothelial cells) stained positive. Conversely, a positive result (retained expression) was interpreted if the tumor cells showed the same intensity staining as the internal positive control cells. If the tumor showed a weak intensity of staining, significantly lower than the normal tissue, the result was interpreted as partial loss of expression.

## Results

### Mutation of SDHA is a recurrent event in young adults with KIT and PDGFRA WT GIST

**Figure 1:**
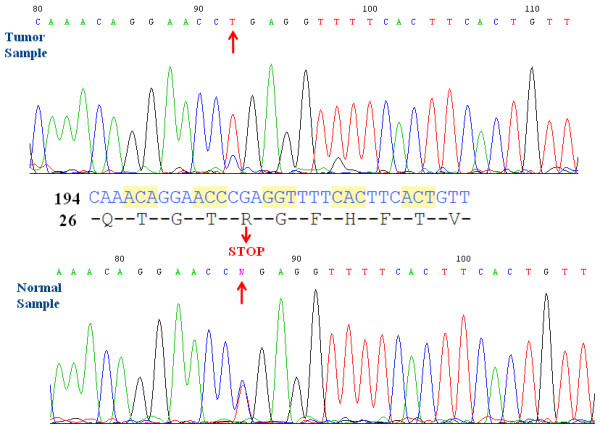
**Sanger sequencing validated a nonsense SDHA exon 2 p.Arg31X mutation in both tumor and normal DNA.** The SDHA sequence electropherogram of the tumor DNA contained predominantly the mutated allele (T), indicating relative loss of the wild-type SDHA allele, compared to the normal DNA.

Massive parallel next-generation sequencing of six cases of WT GIST (five pediatric and one young adult) revealed that the GIST from the young adult patient (22 year-old man, with multinodular gastric lesions and multiple liver metastases) carried a C to T transition at nucleotide 206 in SDHA exon 2, a nonsense mutation resulting in the replacement of arginine with a stop codon at residue 31 of SDHA, causing truncation of the peptide chain at residue 30 (p.Arg31X) (Figure 1). This result was then validated by targeted SDHA exon 2 Sanger sequencing from the DNA isolated from both tumor and normal tissue, in keeping with a germline mutation. Of note, the SDHA sequence electropherogram of the normal DNA revealed equivalent proportion of the wild-type and the mutated allele (T), whereas tumor DNA contained predominantly the mutated allele (T), indicating relative loss of the wild-type SDHA allele. This patient is alive with disease 66 months after the initial diagnosis, and was treated with multiple kinase inhibitors with marginal responses, including imatinib, sunitinib, sorafenib and sirolimus, and being presently on regorafenib.

**Figure 2:**
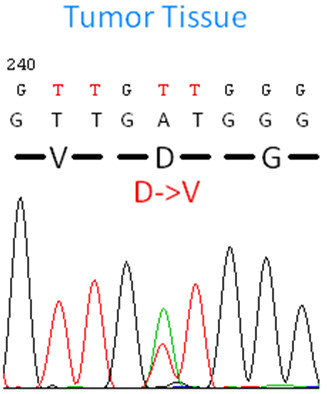
**ABI sequencing showing a somatic mutation in SDHA exon 2 p. D38V.** The normal DNA extracted from this case showed a wild-type sequence for this locus.

Since two prior reports also identified mutations in SDHA exons 9 and 13 [16,17] in WT GIST, we performed targeted SDHA exons 2, 9 and 13 sequencing in 11 additional cases of pediatric and young adult WT GIST. By this method, another young adult WT GIST (26 year-old woman with bulky intra-peritoneal and liver metastatic disease) was identified to harbor a missense mutation in SDHA exon 2 (p. D38V)(Figure 2). In this latter case, the mutation was observed only in the tumor and not in the normal tissue DNA tested. This patient was alive with disease 15 years after the initial diagnosis, preceding the availability for targeted therapy. No additional mutations in SDHA exons 9 or 13 were identified.

### Mutation of SDHA in WT GIST is associated with concomitant loss of both SDHA and SDHB proteins expression

**Figure 3:**
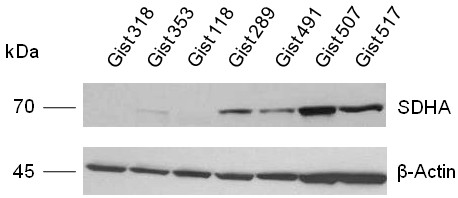
**Western blot showing loss of SDHA expression in the young adult GIST carrying a germline SDHA exon 2 mutation p.Arg31X (GIST318, primary gastric tumor; GIST353, liver metastasis), as well as in the young adult GIST carrying a somatic SDHA exon 2 mutation (GIST118, peritoneal metastasis).** Remaining WT GIST (GIST#289, 491, 507, 517) lacking SDHA mutations showed preserved SDHA protein expression.

**Figure 4:**
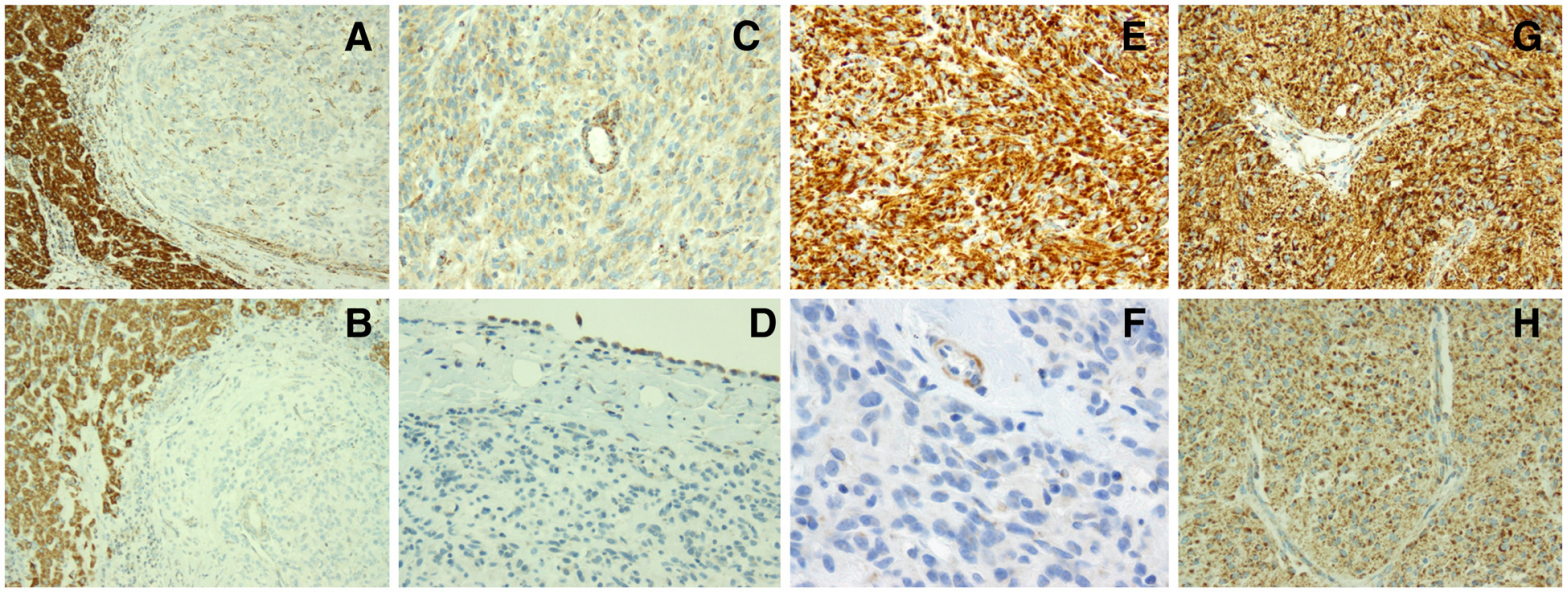
**Immunohistochemistry showed concomitant loss of SDHA and SDHB expression in SDHA-mutant GIST.** Complete loss of expression for SDHA (**A**) and SDHB was noted in the germline SDHA-mutant GIST (**B**), while normal liver showed preserved reactivity. In the somatic SDHA-mutant GIST there was partial loss of SDHA expression (**C**), while SDHB staining was absent (**D**). SDHA expression was retained in a WT pediatric GIST (**E**), while SDHB reactivity was lost (**F**, with internal positive control). In the control KIT exon 11-mutant GIST from a young adult both SDHA (**G**) and SDHB protein expressions were preserved (**H**).

To confirm the functional impact of SDHA mutation, we assessed SDHA protein expression by western blotting in seven WT GISTs, including three samples from the two patients with SDHA mutation and four without SDHA mutation (Figure 3). We found that SDHA expression was absent in the two cases harboring a mutation of SDHA and present in the other cases. In addition, immunohistochemistry for SDHA and SDHB was performed. The WT GIST associated with a germline SDHA mutation showed complete loss of both SDHA and SDHB protein (Figure 4 A,B), while the tumor with a somatic, heterozygous SDHA mutation showed significant decreased in SDHA immuno-expression, as well as complete loss of SDHB (Figure 4 C,D). In contrast, strong and diffuse SDHA reactivity was present in all WT pediatric and young adult GIST tumors tested without detectable SDHA mutations, which matched with a complete loss of SDHB expression (Figure 4 E,F). Furthermore, both SDHA and SDHB expression was preserved in a control case of a young adult GIST carrying a KIT exon 11 deletion (Figure 4 G,H).

## Discussion

The dysregulation of metabolism in cancer has been established for over 80 years. Indeed, one of the first identified biochemical hallmarks of cancer cells was a shift in glucose metabolism from oxidative phosphorylation to aerobic glycolysis [20]. This metabolic conversion was considered for a long time a consequence rather than a cause of cancer. However, this vision has been recently challenged by the finding that a significant proportion of familial and apparently sporadic paraganglioma and pheochromocytoma are related to germline somatic mutation of genes encoding proteins of SDH complex II [21-25]. This complex is a membrane-bound enzyme complex linked to the respiratory chain and a member of the Krebs cycle. It consists of 4 subunits: the flavoprotein subunit (SDHA), the iron-sulfur protein subunit (SDHB), and the integral membrane protein subunits (SDHC and SDHD). Mutations of one of the gene encoding these subunits impair the activity of this complex and lead to the stabilization and activation of HIF-1a, which in turn activates cell proliferation and angiogenesis [12-15].

In addition to paragangliomas and pheochromocytomas, a number of other solid tumors have been associated with mutations in genes encoding the succinate dehydrogenase complex (SDH) complex II. These include gastrointestinal stromal tumors (GIST) [11,16], renal tumors [26], thyroid tumors [26-28], testicular seminoma [29]. The best known association between SDH complex II germline mutations and other tumors is represented by the Carney–Stratakis syndrome (or dyad) which is characterized by the occurrence of KIT and PDGFRA WT GIST and paraganglioma. This syndrome is associated with germline point mutations or large deletions of the genes encoding the SDHB, SDHC or SDHD subunits [30]. Strikingly, inactivating germline mutations in SDHB or SDHC genes have been also identified in sporadic WT GISTs occurring in patients without a personal or family history of paraganglioma [11].

The SDHA gene encodes the major catalytic subunit of the succinate dehydrogenase complex II. Germline mutations in SDHA are associated with neurodegenerative diseases such as an early-onset encephalopathy, known as Leigh syndrome [31-34] and a late-onset optic atrophy, ataxia and myopathy [35]. Until recently, no genetic link between *SDHA* and cancer could be established. However, two recent studies allowed the identification of SDHA germline mutations in at least 3% patients with apparently sporadic cases of paraganglioma or pheochromocytoma [36]. Interestingly, four cases of sporadic *KIT* and *PDGFRA* WT GIST occurring in one pediatric and three young adult patients have also been associated with germline mutation of *SDHA*[16,17]. In the present study, we investigated a series of 17 apparently sporadic and Carney’s triad-related *KIT* and *PDGFRA *WT GIST for *SDHA* mutations and found an additional two cases with mutations in this gene. These were exclusively present in apparently sporadic cases occurring in young adults. The p.Arg31X *SDHA *germline mutation identified in our study leads to a truncated protein [16]. An identical mutation has been previously reported in four Dutch patients with paraganglioma and in one young adult patient with sporadic WT GIST [16,36]. The second *SDHA* mutation identified in our study (p.D38V) has been reported as a single nucleotide polymorphism. However, none of the other 16 GIST cases tested showed this change and this mutation was found only in the tumor DNA, but not in corresponding normal DNA of the patient. Furthermore, this tumor showed significant loss of SDHA protein expression by both western blot and IHC, suggesting a functional impact of this genetic alteration. But since only one source of normal DNA was analyzed in this patient, we cannot formally exclude the possibility of germline mosaicism. Moreover, since we have sequenced only *SDHA* exons 2, 9 and 13, we cannot exclude also the presence of a germline mutation in one of the other exons.

By performing western blotting, we identified a loss of SDHA protein expression in the two mutated cases whereas expression was retained in the non-mutated cases. This result was expected in the tumor with the p.Arg31X mutation because this mutation leads to a truncated SDHA protein. Although the p.D38V missense mutation does not lead to a truncated protein, the SDHA expression was significantly decreased by IHC and not detected by Western blot. This result can be explained by a conformational change of the mutated SDHA protein compromising the antigenic epitope for the antibody. Another explanation is that the p.D38V mutation leads to SDHA protein instability. As indicated above, since only the hot spot exons 9 and 13 of *SDHA* gene were investigated in this case, we cannot exclude the possibility of a germline mutation in a different exon, in keeping with a ‘two-hit’ mechanism of loss of function, implicated in most other SDH-deficient neoplasias. Further investigations are needed to address this point.

The persistent expression of SDHA protein in *SDHA* non-mutated GIST is in accordance with previous studies which showed consistent SDHA protein expression in SDHB-, and SDHD-mutated paraganglioma [25,36]. However, since we have sequenced only exons 2, 9, 13, we cannot exclude the unlikely possibility of a *SDHA *mutation even in cases showing SDHA protein expression. Previous studies demonstrated that SDHB-, SDHC-, and SDHD-related paragangliomas and GIST all show loss of SDHB immunohistochemical expression [11,36]. It was suggested that absence of functional SDHC or SDHD leads to impairment of complex II formation and degradation of SDHB. Our results, showing absence of SDHB expression in *SDHA*-mutated GIST, are in accordance with this explanation. In contrast, whereas SDHB expression was not detected in all WT GIST included in our series, all these tumors (except the two with a mutation of the *SDHA* gene) displayed expression of SDHA. These findings suggest that the SDHB protein is degraded when the succinate dehydrogenase complex II is disrupted, whereas the SDHA protein remains intact.

By pooling our results with those of previous studies [11,16,17,37], it appears that the majority of mutations of genes encoding subunits of the SDH complex II identified in apparently sporadic *KIT* and *PDGFRA* WT GIST occurred in young adults (9 out of 13 patients). However, the majority of sporadic or syndromic *KIT* and *PDGFRA* WT GIST, occurring in the pediatric or young adult setting, display loss of SDHB protein [10], suggesting that defects in cellular respiration is a crucial event even in cases without mutation of the succinate dehydrogenase complex II. Therefore, further investigation are needed to identify the mechanism involved in the alteration of the succinate dehydrogenase complex II function in cases without mutation of SDHA, -B, -C or -D.

## Conclusion

In conclusion, this study provides additional evidence that *SDHA *is another important gene involved in the tumorigenesis of a subset of GISTs lacking *KIT* or *PDGFRA* mutation. Although the number of identified mutation carriers is still low, current observations suggest that mutations of the succinate dehydrogenase complex II are more particularly associated with KIT and PDGFRA WT GIST occurring in young adults, outside the Carney’s triad trait. Although pediatric GIST consistently display alterations of SDHB protein expression, further molecular studies are needed to identify the crucial genetic events involved in their tumorigenesis. Genetic screening for *SDHB*, *C *and D germline mutations is recommended for patients with paraganglioma/pheochromocytoma and SDH deficient GISTs. At the time of this writing, it remains uncertain whether patients with SDHA-deficient GIST are also at increased risk for the tumors associated with SDHx germline mutation. The penetrance of *SHDA* mutations is also unknown. Therefore, further investigations are needed to clarify the clinical significance of a *SDHA* germline mutation and its impact in terms of genetic counseling.

## Competing interests

The authors declare that they do not have any competing interests.

## Authors’ contributions

AI, CLC, and YSS performed the molecular genetic analysis. AI and CRA wrote the manuscript. SS, PB, RPD and MPL participated in project design and provided clinical input. NS run the bioinformatic data analysis. All authors read and approved the final manuscript.

## Pre-publication history

The pre-publication history for this paper can be accessed here:

http://www.biomedcentral.com/1471-2407/12/408/prepub
